# Comparison of Shifts in Skeletal Muscle Plasticity Parameters in Horses in Three Different Muscles, in Answer to 8 Weeks of Harness Training

**DOI:** 10.3389/fvets.2021.718866

**Published:** 2021-10-18

**Authors:** Constance de Meeûs d'Argenteuil, Berit Boshuizen, Carmen Vidal Moreno de Vega, Luc Leybaert, Lorie de Maré, Klara Goethals, Ward De Spiegelaere, Maarten Oosterlinck, Cathérine Delesalle

**Affiliations:** ^1^Department of Translational Physiology, Infectiology and Public Health, Research Group of Comparative Physiology, Faculty of Veterinary Medicine, Research Group of Comparative Physiology, Ghent University, Merelbeke, Belgium; ^2^Wolvega Equine Hospital, Oldeholtpade, Netherlands; ^3^Department of Basic and Applied Medical Sciences, Faculty of Medicine and Health Sciences, Ghent University, Ghent, Belgium; ^4^Department of Veterinary and Biosciences, Faculty of Veterinary Medicine, Research Group Biometrics, Ghent University, Merelbeke, Belgium; ^5^Department of Morphology, Imaging, Orthopedics, Rehabilitation and Nutrition, Faculty of Veterinary Medicine, Ghent University, Merelbeke, Belgium; ^6^Department of Large Animal Surgery, Anaesthesia and Orthopaedics, Faculty of Veterinary Medicine, Ghent University, Merelbeke, Belgium

**Keywords:** exercise, metabolism, muscle fiber typing, capillarization, horse (Equus caballus), mitochondria, muscle plasticity, fuel

## Abstract

Training-induced follow-up of multiple muscle plasticity parameters in postural stability vs. locomotion muscles provides an integrative physiological view on shifts in the muscular metabolic machinery. It can be expected that not all muscle plasticity parameters show the same expression time profile across muscles. This knowledge is important to underpin results of metabolomic studies. Twelve non-competing Standardbred mares were subjected to standardized harness training. Muscle biopsies were taken on a non-training day before and after 8 weeks. Shifts in muscle fiber type composition and muscle fiber cross-sectional area (CSA) were compared in the m. pectoralis, the m. vastus lateralis, and the m. semitendinosus. In the m. vastus lateralis, which showed most pronounced training-induced plasticity, two additional muscle plasticity parameters (capillarization and mitochondrial density) were assessed. In the m. semitendinosus, additionally the mean minimum Feret's diameter was assessed. There was a significant difference in baseline profiles. The m. semitendinosus contained less type I and more type IIX fibers compatible with the most pronounced anaerobic profile. Though no baseline fiber type-specific and overall mean CSA differences could be detected, there was a clear post-training decrease in fiber type specific CSA, most pronounced for the m. vastus lateralis, and this was accompanied by a clear increase in capillary supply. No shifts in mitochondrial density were detected. The m. semitendinosus showed a decrease in fiber type specific CSA of type IIAX fibers and a decrease of type I fiber Feret's diameter as well as mean minimum Feret's diameter. The training-induced increased capillary supply in conjunction with a significant decrease in muscle fiber CSA suggests that the muscular machinery models itself toward an optimal smaller individual muscle fiber structure to receive and process fuels that can be swiftly delivered by the circulatory system. These results are interesting in view of the recently identified important fuel candidates such as branched-chain amino acids, aromatic amino acids, and gut microbiome-related xenobiotics, which need a rapid gut–muscle gateway to reach these fibers and are less challenging for the mitochondrial system. More research is needed with that respect. Results also show important differences between muscle groups with respect to baseline and training-specific modulation.

## Introduction

Skeletal muscles are remarkable in adapting to certain stimuli, such as exercise (1). The muscular compartment represents ±40 up to 57% of total body mass, depending on animal species and breed, which implies that muscular plasticity has a crucial impact on functionality, metabolism and performance capacity of the whole-body system (1–3). It also implies that shifts that take place at the level of certain specific features of the muscular compartment can be viewed as a marker for occurrence of metabolic shifts in answer to training. Several of these markers have been studied in the past, either alone or combined, such as shifts in muscle fiber type composition, muscle fiber cross-sectional area (CSA), mitochondrial density and capillarization (4–7).

Muscle fibers are generally classified as either type I fibers (slow oxidative), type IIA fibers (fast oxidative-glycolytic) or type IIX fibers (fast glycolytic), co-existing with a wide array of transitional hybrid fiber types, such as type I/IIA and type IIA/IIX (8). In horses, the type IIA fiber is the predominant type, followed by the type IIX fiber and the type I fiber (9–12). In general, training can shift muscle fibers from type IIX to the transitional hybrid fiber type IIAX, subsequently to IIA and finally to type I and vice versa (13, 14). Besides shifts in muscle fiber type composition, fiber type specific CSA and mean CSA shifts across all fiber types occur in answer to training. Both fiber type composition and fiber size provide important information on main performance capacity, such as force generation, muscle fiber conduction velocity, and endurance capacity in different species, such as horses and humans (15, 16). It has been shown, for example, that muscle fiber type composition and CSA are directly related to the performance capacity of human endurance athletes (12, 17). Muscle fiber CSA positively correlates with glycogen storage capacity (18), and thus an anaerobic fast-twitch muscle fiber profile (19, 20). Aerobic exercise, on its turn, induces muscle hyperplasia and decreases the muscle CSA, resulting in a smaller distance for oxygen diffusion and swift fuel transport into muscle fibers (21, 22).

The change in muscular capillary supply in answer to training is deemed to be an important plasticity parameter (19). The baseline muscular capillarization level depends on different parameters such as muscle fiber type, muscle fiber CSA, oxidative capacity, and metabolic environment (23, 24). Physiologically, it is interesting to notice that in both humans (25–28) and horses (4, 24, 29–31), baseline muscular capillarization level increases in answer to training, no matter what type of training is imposed (either aerobic endurance vs. anaerobic resistance/sprint type of training).

Finally, also mitochondrial plasticity occurs following training (32, 33). However, unlike capillarization, the observed mitochondrial plasticity seems to depend on the type of imposed exercise (34). On top of that, both an acute (following a few high-intensity exercise sessions) (35) and a long-term (following several weeks to months of training) (36–38) mitochondrial plasticity can occur. Mitochondria are pivotal for oxidative metabolism and adapt to different types of exercise by both morphological and functional modulations (36, 39, 40). Morphological adaptations comprise changes in mitochondrial density, and at the ultrastructural level, changes in the amount and length of mitochondrial cristae and density of mitochondrial DNA. Functional adaptations encompass changes in mitochondrial enzymatic activity [e.g., citrate synthase (CS), succinate dehydrogenase activity, monocarboxylate transporter 1 (MCT1), basigin, 3-hydroxyacyl-CoA dehydrogenase (HAD), hexokinase (HK) and lactate dehydrogenase (LDH)] or changes in mitochondrial respiration (41–43). Endurance exercise, for example, increases mitochondrial density, whereas strength training decreases mitochondrial density by inducing muscle fiber hypertrophy and mitochondrial fragmentation (44, 45). In horses, only a limited number of studies on the effects of different types of exercise on muscle mitochondrial density are available (4, 23, 46–48).

Skeletal muscles contain two distinct mitochondrial subpopulations: intermyofibrillar mitochondria (IMF), distributed between myofibrils and subsarcolemmal (SS) mitochondria situated underneath the sarcolemmal membrane (36, 43). Both specific populations are reported to react differently in answer to training (42, 43, 49). Oxidative or slow-twitch fibers have a high mitochondrial density, predominantly in the subsarcolemmal region, whereas fast-twitch fibers have a low mitochondrial density that appears more fragmented throughout the muscle fiber (50). Mitochondrial populations form a dynamic network within the muscle fibers, allowing them to interconnect *via* fusion and fission (42, 43, 49). Inter-mitochondrial connections expand in answer to aerobic exercise, due to increased fusion between mitochondria of either subtype, i.e., SS or IMF (51).

Longitudinal follow-up of all four muscle plasticity parameters (shifts in muscle fiber type composition, muscle fiber CSA, capillarization, and mitochondrial density) provides a more integrative physiological view on what changes to expect at the level of the muscular metabolic machinery, when compared to focusing on a subset of these plasticity parameters. It can be expected that not all four muscle plasticity parameters show the same expression time profile and thus differences can be expected between these plasticity parameters when it comes to early identification of training adaptation (4, 6).

A study by Tyler et al. reports the longitudinal follow-up of all aforementioned muscle plasticity parameters (fiber type composition, CSA, capillarization, and mitochondrial density) in answer to respectively 7 weeks of endurance training and 8 weeks of high-intensity exercise in Standardbreds, focusing on one muscle: the m. gluteus medius (4). In that study, both capillarization and mitochondrial density showed significant changes in answer to both types of training, whereas no significant changes could be found with respect to shifts in fiber type composition and CSA. Therefore, not all muscle plasticity parameters seem equally discriminative or sensitive when it comes to follow-up of training adaptation. Another study reports the absence of change in mitochondrial density in the m. gluteus medius of Quarter Horses subjected to respectively 9 (46) and 14 weeks (52) of submaximal exercise. However, these researchers did find training-induced increased mitochondrial respiratory activity, expressed by upregulation of OXPHOS complex II activity, and electron transport chain capacity, underlining the fact that mitochondrial functional shifts represent an additional set of possible muscular plasticity parameters (46, 53, 54).

Many training studies have been combining two or three out of the four aforementioned muscle plasticity parameters (5–7), most of them, focusing on the m. gluteus medius, known as an archetype locomotion-function type of muscle group (55, 56).

However, to our knowledge, at this point, no training studies are available involving longitudinal follow-up of all four muscle plasticity parameters in multiple muscles with differing main mechanical function (muscles mainly involved in postural stability vs. muscles involved in locomotion). It can be expected that each type of training modulates a different core set of muscles (55, 56). By involving both “main posture vs. main locomotion directed muscle groups” at the same time, a much more integrative and subtle physiological insight is obtained into how the muscular compartment adapts in response to a certain training type. Depending on the main mechanical function of a certain muscle group, different metabolic challenges are imposed by the applied training type. Obtaining such detailed view on muscular adaptation is crucial for further optimization of existing training protocols and understanding of muscular pathologies.

The aim of the current study was to map out and compare the evolution of four important muscle plasticity parameters in a core set of muscles in answer to training, to obtain a better view on flexibility of these parameters and their expression profile in muscles mainly involved in postural stability vs. muscles involved in locomotion. For this purpose, 12 Standardbred horses were subjected to a harness training protocol of 8 weeks duration.

## Materials and Methods

### Study Setup

Twelve untrained, non-competing Standardbred mares aged between 3 and 5 years were subjected to a standardized training trial (*N* = 12). Horses were comparable in body condition score (5.5 ± 0.70) and body weight (446.5 ± 20.94 kg) and housed at the same training facility in individual boxes with straw bedding. Turn-out was provided on paddocks 2 h a day. Horses were fed the same concentrate feed twice a day, at 8 a.m. and 8 p.m. and had *ad libitum* access to tab water and roughage. Monitoring for vital signs (pulse, temperature, respiratory rate, capillary refilling time, appetite, and stool production) was performed twice a day and horses were allowed 2 weeks of acclimation prior to start of the training trial. All horses remained in good health throughout the study. No adverse events occurred.

### Training Regime

All horses (*N* = 12) were harness trained during eight consecutive weeks by the same experienced driver 4 days a week on the same oval-shaped sand race track, between 9 and 12 a.m., during the cooler European months. Horses were equipped with a GPS tracker and heart rate monitoring system (Polar^®^ Equine H7, Polar Electro Oy, Finland). Each training session consisted of a warming-up period of 10 min jogging (±20 km/h), followed by either 30 min of aerobic training (3 days a week, ± 25 km/h, mean HR < 150 BPM) or interval training (day 4) consisting of 3 × 3 min at high speed (±35 km/h). The track was watered and raked on each occasion, to maximally assure equal study conditions throughout the trial. Muscle biopsies were taken on a resting day before and after the 8-week training period, at the level of the m. pectoralis (expected postural predominant), the m. semitendinosus (expected pure locomotor), and the m. vastus lateralis of the quadriceps femoris (expected locomotor predominant). This study was approved by the Animal Ethics Committee of the Ghent University (EC 2016/40).

### Muscle Biopsies

Briefly, the horses were sedated with detomidine (10 μg/kg bwt) (Detogesic, Vetcare, Finland) and butorphanol (20 μg/kg bwt) (Butomidor, Richter Pharma AG, Wels, Austria). The area was clipped, shaved, and subsequently disinfected. Local anesthetic ointment was applied (Emla 5%, Astra-Zeneca, Rueil-Malmaison, France). After 10 min, local anesthetic (Lidocaine Hydrochloride, Braun, Germany) was injected subcutaneously and a small stab incision was made with a surgical blade number 11. Thereafter, a 14G Bergström needle was inserted into the muscle, until a depth of 4 cm was reached on each occasion. Samples were placed in Tissue-Tek O.C.T compound (Sakura Finetek, Torrance, California) and were immediately snap-frozen in liquid nitrogen-cooled isopentane (Sigma-Aldrich, Dorset, UK) and stored at −80°C until further processing.

### Muscle Fiber Typing

Cryosections of 8 μm were created from the Tissue-Tek embedded samples and were collected onto coated glass slides (Thermo Scientific SuperFrost Plus Adhesion slides, Fisher scientific, Belgium) and stored at −20°C until further processing. Briefly, the sections were air-dried and then blocked for 120 min in 1% bovine serum albumin (BSA) in phosphate buffered saline (PBS) solution at room temperature. The slides were than shortly washed with permeabilization solution (BSA and 0.2% triton in PBS solution). Thereafter, the slides were incubated overnight at −4°C with primary antibodies for the different myosin heavy chains (57) dissolved in 0.5% BSA in PBS, for type I, type IIA, type IIX, and sarcolemma [BA-D5 (type I), dilution 1:400; DSHB, RRID:AB_2235587; SC-71 (type IIA), dilution 1:100; DSHB, RRID:AB_2147165; 6H1 (type IIX), dilution 1:50; DSHB, RRID:AB_1157897 and laminin (sarcolemma), dilution 1:500; Thermo Fisher Scientific Cat: PA1-36119, RRID:AB_2133620]. No controls were applied. After rinsing the slides five consecutive times during 5 min in PBS, and washing them shortly with permeabilization solution, they were incubated with the secondary antibodies dissolved in 0.5% BSA in PBS for 1 h at room temperature for type I, IIA, IIX (dilution 1:300), and sarcolemma (dilution 1:600) [Alexa fluor 488 goat anti-mouse IgG2b (type I), Thermo Fisher Scientific Cat: A-21141, RRID:AB_2535778; Alexa fluor 350 goat anti-mouse IgG1 (type IIA), Thermo Fisher Scientific Cat: A21120, RRID:AB_2535763; Alexa fluor 594 goat anti-mouse IgM (type IIX), Thermo Fisher Scientific Cat: A-21044, RRID:AB_2535713; Alexa fluor 568 goat anti-rabbit IgG (sarcolemma), Thermo Fisher Scientific Cat: A-11011, RRID:AB_143157]. After rinsing the slides five consecutive times during 5 min in PBS, they were air dried and fluorescent mounting medium (Dako, Agilent, S3023) was applied on the slides with a cover glass.

For the capillaries and the mitochondria, the slides were incubated overnight at −4°C with the primary antibodies targeting the Von Willebrand factor (capillaries) (polyclonal rabbit anti-human Von Willebrand factor, dilution 1:1600; Agilent Cat# A0082, RRID:AB_2315602) and COX IV (mitochondria) (anti-COX IV antibody, dilution 1:100; Abcam Cat# ab14744, RRID:AB_301443) diluted in 0.5% BSA in PBS. After rinsing the slides and washing them with permeabilization solution, the sections were incubated for 1 h at room temperature with the secondary antibodies diluted in 0.5% BSA in PBS [Alexa fluor 568 goat anti-rabbit IgG (capillaries), dilution 1:600; Thermo Fisher Scientific Cat# A-11011, RRID:AB_143157 and Alexa fluor 647 goat anti-mouse IgG2a (mitochondria), dilution 1:500; Thermo Fisher Scientific Cat# A-21241, RRID:AB_2535810]. The sections were visualized with a Zeiss Palm Micro Beam fluorescence microscope and pictures were taken with the Zen Blue Pro Software (Zeiss). Fibers were classified as either type I (green), type IIA (blue), type IIX (red), or as hybrid type when staining for more than one myosin heavy chain was present. Total fiber count, fiber type percentages, and CSAs were determined as well as capillary count and mitochondrial intensity using Image Pro v.10 analyzer software (Media Cybernetics, Inc., Rockville, USA). All sections were evaluated in duplicate, with a minimum of 250 fibers per section. The mean CSA of each fiber type was measured, called from now on fiber type specific CSA, as well as the overall mean CSA across all muscle fiber types. The mean CSA of all fibers was calculated for all three muscles in two different ways: one calculating the average of all the CSA obtained for each fiber type that we will refer to as “mean mathematical CSA”, and another method that relies on a count per frame approach to which we will refer as “mean frame CSA”. For the m. semitendinosus, we also calculated for each fiber type and for all fibers the mean minimum Feret's diameter, which is the closest possible distance between the two parallel tangents of a muscle fiber. Capillaries surrounding type I and type IIA fibers were counted (CAF), and capillary density (CD) (number of capillaries/mm^2^) and capillaries per fiber ratio (C/F) (depicting the number of fibers in contact with each capillary) were calculated. CAF and C/F were counted over the whole section, and CD was done by counting and summing four fields of each 250,000 μm^2^, chosen at random, but covering the whole section. For assessment of mitochondrial density, each muscle fiber image was divided into two segments: one from the sarcolemma going 15 μm deeper into the fiber (subsarcolemmal mitochondria) and one from 15 μm deep, to the center of the fiber (intermyofibrillar mitochondria). Mitochondrial density was expressed as the intensity of fluorescence in type I and type IIA fibers and intensity in the SS and the IMF region and the fold change was calculated by dividing mitochondrial density of type I by mitochondrial density of type IIA. To assess mitochondrial density, a minimum of 75 fibers for each fiber type were chosen at random in different fields, but covering the whole section. To look at the effect of training, a ratio between SS mitochondria and IMF mitochondria was calculated as well as the difference in mitochondrial density between type I and type IIA fibers before and after training.

### Statistical Analysis

Different fiber types were counted and classified per type and relative percentages of each type were calculated for each of the muscles. The mean CSA using both methods as well as mean fiber CSA of all types of fibers separately were determined in the three muscles (in μm^2^).

Statistical analysis was performed in R (104). Normality of the data was graphically checked using histograms and Shapiro–Wilk's test. Because normality of the data could not be assumed, data were analyzed using nonparametric tests. The results are given as median (minimum–maximum). Significance was set at *p* < 0.05.

The effect of muscle type (m. pectoralis vs. m. vastus lateralis vs. m. semitendinosus) on the percentages of fiber types, fiber type specific CSA, mean frame CSA, and mean mathematical CSA was analyzed using a Friedman test. If the effect was significant, pairwise comparisons were tested using a Wilcoxon signed rank test at a significance level of 0.017 to account for multiple comparisons (Bonferroni correction for multiple pairwise comparisons: α = 0.05/3).

The effect of training on muscle fiber type composition, fiber type specific CSA, mean frame CSA and mean mathematical CSA per studied muscle, and the fiber type specific minimum Feret's diameter, mean minimum Feret's diameter, capillary, supply and mitochondrial density was analyzed using a Wilcoxon signed rank test.

## Results

### Comparison of Baseline Muscle Fiber Type Composition, Fiber Type Specific CSA, Mean Mathematical CSA, and Mean Frame CSA in the m. pectoralis, m. vastus lateralis, and m. semitendinosus

In the current study, apart from the “pure” fiber types, only hybrid type IIA/IIX (IIAX) fibers were included in the analysis, since the hybrid type I/IIA was only sporadically found (<0.5%).

Only the baseline profiles of fiber types I (*p* = 0.0057) and IIX (*p* = 0.0001) showed significant differences between the studied muscles (Table 1).

**Table 1:** Baseline muscle fiber type composition of the m. pectoralis, the m. vastus lateralis, and the m. semitendinosus.

	**Muscle fiber types (%)**			
**Muscles**	**Type I (%)**	**Type IIA (%)**	**Type IIAX (%)**	**Type IIX (%)**
m. pectoralis	24.7 (21.1–37.5)^*^	52.8 (39.3–68.8)	6.9 (1.4–12.5)	16.6 (2.1–28.8)^*^
m. vastus lateralis	20.4 (3.5–30.2)^*^	54.9 (42.4–66.3)	8.4 (2.1–14.4)	17.5 (4.7–33.2)^*^
m. semitendinosus	13.6 (0.3–30.4)	46.7 (24.7–71.1)	5.4 (0.8–18.7)	34.5 (21.2–62.4)

* *Significantly different from the m. semitendinosus, p < 0.017*.

*Percentages of type I, type IIA, type IIAX, and type IIX are given as median (minimum–maximum)*.

There was a significantly higher percentage of fiber type I content for both the m. pectoralis (*p* = 0.0017) and the m. vastus lateralis (*p* = 0.0081) compared to the m. semitendinosus. A significantly higher percentage of type IIX fibers was present in the m. semitendinosus compared to the m. pectoralis (*p* = 0.0005) and the m. vastus lateralis (*p* = 0.0005). There were no significant differences in percentage of type IIA and IIAX fibers between the three different muscles (Table 1).

No significant difference in fiber type specific CSA of type I, type IIA, and type IIAX could be found between the three muscles before training. Additionally, there was no significant difference observed in baseline mean frame and mean mathematical CSA between the three muscles.

### Training-Induced Shifts in Muscle Fiber Type Composition, Fiber Type Specific CSA, Mean Frame CSA, and Mean Mathematical CSA in the m. pectoralis, the m. vastus lateralis, and the m. semitendinosus

No significant shifts in fiber type composition could be detected in the m. pectoralis and the m. vastus lateralis for either type I, type IIA, type IIAX, or type IIX; however, the m. semitendinosus showed a significant increase in type I fibers in answer to training [from 13.6 (0.1–30.4) to 26.9% (6.9–34.2)] (*p* = 0.0034).

Fiber type specific CSA showed evolution in answer to training, but not in all studied muscles. No change in fiber type specific CSA of type I, IIA, IIAX, and IIX could be detected in the m. pectoralis in answer to training. The m. vastus lateralis, however, showed much more plasticity: there was a significant decrease in fiber specific CSA of all fiber types in answer to 8 weeks of harness training (type I: *p* = 0.0046; type IIA: *p* = 0.0061; type IIAX: *p* = 0.0134; and type IIX: *p* = 0.0081). Also, the m. semitendinosus showed plasticity, though to a much lesser extent. In that muscle, there was only a significant decrease of the type IIAX fiber type specific CSA (*p* = 0.0342) (Table 2).

**Table 2:** Comparison of fiber type specific cross-sectional area (CSA) of the different muscle fiber types in the m. pectoralis, the m. vastus lateralis, and m. semitendinosus before training (untrained) and after 8 weeks of harness training (trained).

		**Muscle fiber type specific CSA** **(in ***μ***m^2^)**			
**Muscles**	**Training state**	**Type I (μm^2^)**	**Type IIA (μm^2^)**	**Type IIAX (μm^2^)**	**Type IIX (μm^2^)**
m. pectoralis	Untrained	4,517 (2,131–10,103)	5,685 (3,307–12,511)	5,921 (3,590–15,789)	5,684 (3,407–15,738)
	Trained	3,678 (2,231–5,474)	5,673 (3,067–9,768)	4,935 (2,760–11,931)	5,549 (2,216–8,636)
m. vastus lateralis	Untrained	3,108 (2,314–4,449)	5,670 (4,096–8,493)	6,165 (4,562–10,513)	5,800 (4,528–9,289)
	Trained	2,084 (1,627–3,331)^**^	4,563 (3,063–6,868)^**^	5,309 (3,235–7,350)^*^	4,585 (2,549–7,908)^**^
m. semitendinosus	Untrained	5,184 (2,420–9,917)	5,383 (3,295–10,689)	7,077 (3,701–13,289)	8,489 (5,484–12,159)
	Trained	4,025 (2,922–6,672)	4,959 (2,947–10,452)	5,856 (3,482–10,708)^*^	5,795 (3,439–10,161)

* *Significantly different from untrained, p < 0.05*.

** *Significantly different from untrained, p < 0.01*.

*Fiber type specific CSA expressed in μm^2^ are given as median (minimum–maximum)*.

**Figure 1:**
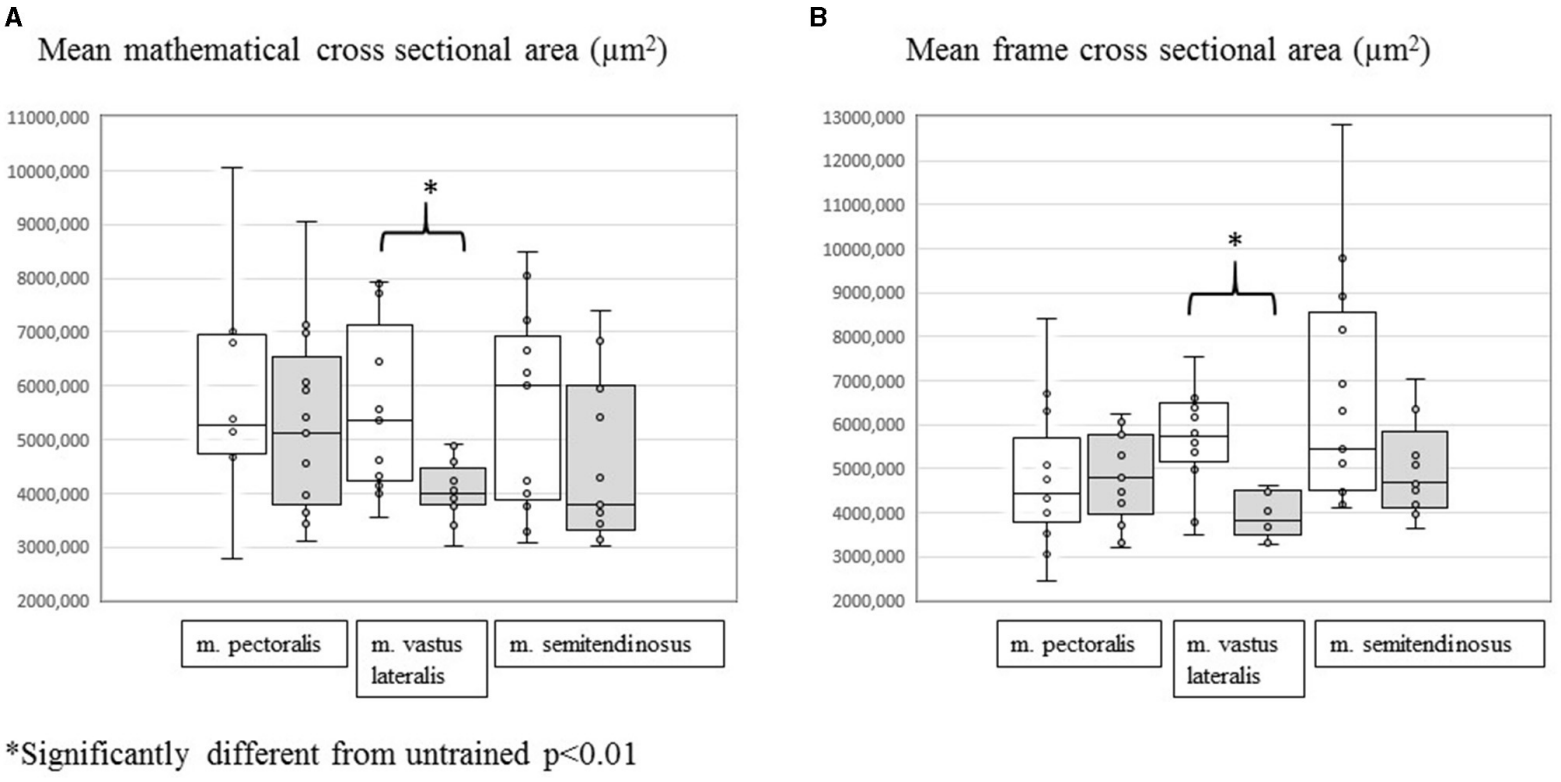
Evolution of mean mathematical CSA **(A)** and mean frame CSA **(B)** in the m. pectoralis, m. vastus lateralis, and m. semitendinosus before (white plots) and after (gray plots) 8 weeks of harness training. *Significantly different from untrained, *p* < 0.01.

When comparing mean mathematical and mean frame CSA before and after training per studied muscle group, the m. vastus lateralis showed significant training-induced shifts. There was a significant decrease in both the mean frame CSA [from 5,740 (3,487–8,568) μm^2^ to 3,818 (3,281–4,640) μm^2^] (*p* = 0.0007) and the mean mathematical CSA [from 5,351 (3,562–7,929) μm^2^ to 3,985 (3,025–4,922) μm^2^] (*p* = 0.0046) in answer to training. No significant changes were recorded for the m. pectoralis and the m. semitendinosus (Figure 1).

### Training-Induced Shifts in the Fiber Type Specific Minimum Feret's Diameter and Mean Minimum Feret's Diameter in the m. semitendinosus

There was a significant effect of harness training on the fiber type specific minimum Feret's diameter in the m. semitendinosus of fiber type I (*p* = 0.0214), which shows a decrease in diameter after 8 weeks of training. Fiber type IIA, IIAX, and IIX specific minimum Feret's diameter did not show any significant change in answer to training (Table 3).

**Table 3:** Comparison of fiber type specific and mean minimum Feret's diameter in the m. semitendinosus before training (untrained) and after 8 weeks of harness training (trained).

**Training state**	**Minimum Feret's diameter** **(***μ***m)**				
	**Type I (μm)**	**Type IIA (μm)**	**Type IIAX (μm)**	**Type IIX (μm)**	**Mean (μm)**
Untrained	66.76 (49.97–94.33)	66.99 (52.58–92.35)	78.83 (56.96–101.01)	85.40 (62.79–99.46)	73.27 (58.15–96.32)
Trained	56.23 (49.26–68.88)^*^	65.19 (49.71–78.31)	72.70 (54.63–100.59)	65.59 (54.45–94.83)	63.06 (58.27–82.14)^*^

* *Significantly different from untrained, p < 0.05*.

*Minimum Feret's diameter is expressed in μm and results are given as median (minimum–maximum)*.

Across all fiber types, there was a significant training-induced decrease in the mean minimum Feret's diameter in the m. semitendinosus after 8 weeks of training (*p* = 0.0398).

### Comparison of Mean Mathematical CSA and Mean Frame CSA Across All Three Studied Muscles After 8 Weeks of Harness Training

The mean frame CSA was significantly different between the three muscle groups after 8 weeks of harness training (*p* = 0.0029). The mean frame CSA was smaller in the m. vastus lateralis [3,818 (3,281–4,620) μm^2^] when comparing to the m. pectoralis (*p* = 0.0105) [4,783 (3,209–6,238) μm^2^] and to the m. semitendinosus (*p* = 0.0017) [4,680 (3,642–8,794) μm^2^].

When looking at mean mathematical CSA, no significant difference was observed between the three muscle groups.

### Training-Induced Shifts in Capillary Supply in the m. vastus lateralis

Since no shifts were seen in muscle fiber type composition, or in fiber specific and mean fiber CSA of the m. pectoralis in answer to 8 weeks of harness training, and only few changes were observed in the m. semitendinosus, it was decided to focus on the m. vastus lateralis to further map out changes in capillary supply and mitochondrial density.

Capillary density (1.58-fold change; *p* = 0.0015), the number of capillaries surrounding type I (1.17-fold change; *p* = 0.0068) and type IIA fibers (1.21-fold change; *p* < 0.001), and the capillary-to-fiber ratio (C/F) (1.24-fold change; *p* < 0.001) were all significantly increased in answer to 8 weeks of harness training (Table 4).

**Table 4:** Comparison of capillary density, number of capillaries surrounding type I and type IIA fibers, and capillary-to-fiber ratio before training (untrained) and after 8 weeks of harness training (trained).

**Training status**	**Capillary density (/mm^2^)**	**Capillaries surrounding fiber type**		**Capillaries/fiber ratio**
		**Type I**	**Type IIA**	
Untrained	330 (206–600)	4.79 (3.86–5.68)	6.11 (4.8–7.84)	2.09 (1.69–2.79)
Trained	521 (437–614) ^*^	5.60 (4.28–7.0) ^*^	7.39 (5.56–9.34) ^*^	2.59 (1.99–3.37) ^*^

* *Significantly different from untrained, p < 0.01*.

*Results are given as median (minimum–maximum)*.

### Training-Induced Shifts in Mitochondrial Density in the m. vastus lateralis

Significant differences in baseline fiber type specific mitochondrial subpopulations could be detected in the m. vastus lateralis (*p* < 0.001). However, no significant training-induced shifts could be detected with respect to general and subpopulation specific mitochondrial density in the m. vastus lateralis.

No significant changes in mitochondrial density could be detected in answer to training (Table 5). The ratio between SS and IMF mitochondria in type I fibers and type IIA fibers did not change in answer to harness training. When looking at the effect of harness training on mitochondrial density between type I and type IIA fibers, no significant effect could be detected for either SS or IMF mitochondria (Table 5).

**Table 5:** Ratio between subsarcolemmal and intermyofibrillar mitochondrial density in type I and type IIA fibers and ratio between type I and type IIA fibers of subsarcolemmal and intermyofibrillar mitochondria before training (untrained) and after 8 weeks of harness training (trained).

	**Subsarcolemmal/intermyofibrillar mitochondria**		**Type I fibers/type IIA fibers**	
**Training status**	**Type I**	**Type IIA**	**Subsarcolemmal**	**Intermyofibrillar**
Untrained	1.42 (1.27–1.75)	1.73 (1.53–2.29)	1.55 (1.19–2.22)	1.84 (1.44–2.77)
Trained	1.53 (1.27–1.93)	1.92 (1.39–2.45)	1.51 (1.16–1.94)	2.01 (1.37–2.32)

*Results are given as median (minimum–maximum)*.

Before training, both SS (*p* < 0.001) and IMF (*p* < 0.001) mitochondria were significantly more represented in type I fibers when compared to type IIA fibers (1.44- and 1.75-fold, respectively), and this was still the case after training (1.63- and 1.98-fold, respectively) (*p* < 0.001). Note that type I fibers contain twice as many IMF mitochondria as type IIA fibers. Furthermore, the mitochondrial density of the SS region was higher than the density at the IMF region, and this was the case in both fiber types before (1.52-fold for type I and 1.85-fold for type IIA; both *p* < 0.001) and after 8 weeks of harness training [1.49-fold for type I (*p* < 0.001) and 1.80-fold for type IIA (*p* < 0.001)] (Table 6).

**Table 6:** Comparison of subsarcolemmal and intermyofibrillar mitochondrial density, expressed as intensity of fluorescence, between type I and type IIA fibers before training (untrained) and after 8 weeks of harness training (trained).

	**Untrained**		**Trained**	
**Fiber type**	**Subsarcolemmal region**	**Intermyofibrillar region**	**Subsarcolemmal region**	**Intermyofibrillar region**
Type I	59.91 (10.27–109.39)	39.48 (7.01–86.26)	54.33 (42.50–75.88)	36.48 (21.96–59.42)
Type IIA	41.67 (7.67–49.14)^*^	22.49 (3.81–31.13)^*^	33.24 (25.05–54.95)^*^	18.41 (12.47–39.44)^*^

* *Significantly different from type I, p < 0.01*.

*Results are given as median (minimum–maximum)*.

## Discussion

To the best of our knowledge, this is the first training study to monitor four different muscle plasticity parameters (muscle fiber type composition, fiber type specific and mean fiber CSA, capillary supply, and mitochondrial density) in three muscles (posture vs. locomotor predominant). This study approach has enabled a better understanding of which muscle plasticity parameters change most rapidly in response to harness training. By combining locomotion dominant vs. posture dominant muscles, the observed changes can also be more reliably translated to the metabolic metamorphosis undergone by the muscle group involved. Results of the study show that of all four studied muscle plasticity parameters, the mean CSA, and the capillary supply show pronounced shifts in answer to harness training and thus can function as early indicators of training adaptation. With that respect, the m. vastus lateralis showed most plasticity, showing a decrease in CSA of all studied muscle fiber types, followed by the m. semitendinosus, showing only a decrease in CSA of the type IIAX fibers. Both, the m. vastus lateralis and m. semitendinosus are suitable for repeated harvesting of muscle biopsies without the occurrence of complications.

When looking into scientific literature, a large body of research has primarily focused on the m. gluteus medius (4, 7, 58, 59), and only a few studies have included more than one muscle (60–63); in some cases, even a combination of postural and locomotor muscles have been included (60, 64), but never focusing on all four muscle plasticity parameters at the same time.

With respect to sample size, the involvement of 12 horses seems limited compared to rodent training studies; however, because of the intensive management and costs associated with equine training studies, it is practically not realizable to include many more horses in a highly standardized training study. When looking in literature, most existing training studies performed on horses include five to maximum 10 horses (31, 65).

Hodgson and coworkers analyzed muscle fiber type composition and capillarization, together with mitochondrial enzymatic activity in the m. gluteus medius of seven horses before and after 9 months of endurance training (5). They found an increase in the percentage of type IIX fibers showing an increased concentration of mitochondria along with an increase in the number of capillaries surrounding these fibers (5). An increase in mitochondrial enzymatic activity was already observed after 3 months of training (5). Karlström et al. studied the training-induced shift in fiber type composition and CSA in the m. gluteus medius of 18 racehorses in order to compare between young (2 years old) and more aged trained horses (4–8 years old) without subjecting these horses to a standardized training protocol (6). They found in the more aged and trained horses a larger CSA for fiber type I and a smaller CSA for fiber type IIX, as well as a more numerous representation of fiber type IIA and fewer fiber type IIX compared to younger horses. After performing NADH tetrazolium reductase staining of muscle sections to measure dehydrogenase activity, an age- and training-dependent increase of the oxidative capacity of type IIX fibers was also observed (6). Revold et al. analyzed training-induced shifts in muscle fiber type composition and CSA together with metabolic changes in the m. gluteus medius of nine young Coldblooded trotter horses (7). Despite the lack of standardized training, these researchers found a training-dependent decrease in fibers of type IIX and an increase in fibers of type IIAX, together with an increase in cytoplasmic MCT 1 and basigin expression in fibers of respectively type IIAX and IIA, and an increase in CS and HAD of the m. gluteus medius (7).

Studies that have involved multiple muscle groups predominantly focus on comparing muscle plasticity parameters between muscle groups under baseline conditions (60, 61). For instance, Karlström et al. explored the baseline differences in fiber type composition, capillarization, and enzymatic profile between 10 locomotor muscles and 3 non-locomotor muscles in three Swedish Standardbred untrained horses (60). In that study, it has been shown that non-locomotor muscles such as the m. masseter and diaphragm have a high percentage of type I fibers (70–100%) and almost no type IIX fibers (<1%), and this was accompanied by a high oxidative and low glycolytic capacity, in opposite of what was seen in locomotor muscles such as m. vastus lateralis and semitendinosus. Regarding locomotor muscles, the m. vastus lateralis showed a higher percentage of type I fibers and a lower percentage of type IIX fibers compared to the m. semitendinosus (60). Essén and partners described the effect of age associated with unspecified training in 55 Standardbred horses divided into five different age groups, chosen to represent ages of interest according to muscle growth and activity (61). They analyzed the baseline fiber type composition of four different locomotor muscles: the m. gluteus medius, the m. vastus lateralis, the m. semitendinosus, and the m. triceps brachii. They found a predominant presence of fibers of type II in all horses, with the m. semitendinosus having a higher proportion of this fiber type compared to the other muscles. The m. gluteus medius showed a higher type I/type II ratio in older horses compared to foals and the m. vastus lateralis had a higher content in type IIA compared to other muscles, whereas the m. semitendinosus presented a higher content of type IIX fibers. The glycolytic capacity was found to be higher in the m. gluteus medius and the m. semitendinosus, but with age, a higher oxidative capacity was seen to develop in these two muscles. This study used the ATPase staining method instead of the more widely used myosin heavy chain (MHC) method (61). In a different study, Grotmol and his team also examined the baseline fiber type composition and also the spatial distribution of muscle fibers in different muscle layers of the m. gluteus medius and the m. semitendinosus in four American Standardbred horses that underwent unspecified racetrack training (63). The results showed a predominance of type IIX fibers at the surface of the fascicles, and of types I and IIA in the more central parts. This type II/type I difference between the superficial and the deeper sites was especially more pronounced in the m. gluteus medius (63). In a different study, Kawai et al. described the baseline muscle fiber type composition and metabolic properties of 46 different muscles from the whole body in six Thoroughbred horses, including the m. pectoralis profundus, the m. vastus lateralis, and the m. semitendinosus. In the hindlimb muscles, the fiber type IIA was predominant, and the m. vastus lateralis was one with the highest proportion of this fiber type. Additionally, the mean percentage of type IIX fibers in the m. gluteus medius was the highest compared to the rest of the muscles in this group. The hindlimb group had overall the lowest percentage of type I and IIA fibers (64). Additionally, only a few studies have involved the comparison of two locomotor muscles before and after training (61–63). For example, Dingboom et al. studied in 38 young Dutch Warmblood foals divided into three exercise groups (box-rest, training, and pasture) the difference in muscle fiber type composition of the deep m. gluteus medius and the m. semitendinosus. In that study, no effect of exercise could be seen; however, changes in composition could be seen with age. From 22 to 48 weeks, a decrease in type IIX fibers was observed in the m. semitendinosus, whereas an age-dependent increase of type I and IIA fibers, as well as an increase in type IIAX fibers, was found in the m. gluteus medius (62).

In the current study, the multimodal approach allowed for obtaining a view on and comparison of baseline muscle fiber type composition and fiber type specific CSA, mean mathematical CSA, and mean frame CSA between all three studied muscle groups. With that respect, no baseline CSA differences could be detected. On the other hand, significant differences were detected in the baseline representation of type I and type IIX fibers, while no differences were found when looking at type IIA fibers, the predominant fiber type in horses.

Both the m. pectoralis and the m. vastus lateralis harbored significantly more type I fibers, when compared to the m. semitendinosus (66). The m. semitendinosus on its turn, showed significantly more type IIX fibers when compared to both the m. pectoralis and the m. vastus lateralis. This is in accordance with previous studies (60, 61, 63, 64). Based on the baseline fiber type composition, the most pronounced aerobic metabolic profile could be assigned to the m. pectoralis, the least aerobic to the m. semitendinosus. This is also the first study to show that both the mean mathematical and mean frame CSA is quite comparable between these muscle groups.

It is important to notice that the baseline fiber type composition of the m. vastus lateralis in the current study is comparable with that reported for the m. gluteus medius in Standardbred trotter horses in other studies (4, 67, 68). The ease with which biopsies can be harvested in the m. vastus lateralis is an added value for studies in which a longitudinal follow-up of several weeks to months is performed and in which for example also harvest of acute biopsies (taken immediately after exercise) is involved. Besides that, the m. vastus lateralis is often involved in human muscular studies, which could open up new avenues from a comparative point of view (69–71).

When looking at training adaptation, the m. vastus lateralis showed the most plasticity, which was manifested by a significant decrease in the fiber type specific CSA of all studied muscle fiber types and a significant decrease of the mean CSA, and this was applicable for both the frame and mathematical methods. Training adaptation also occurred in the m. semitendinosus, but to a much lesser extent. In that muscle, there was a significant increase in the percentage of type I fibers and a decrease in the fiber type specific CSA of type IIAX fibers. No significant change in the mean CSA of all fibers was detected with either method. The m. pectoralis showed no training adaptation with the applied histological methods. So, despite the fact that baseline fiber type specific CSA and mean CSA did not differ between all three studied muscle groups at the start, a clear training-induced response was visible, and this was the muscle plasticity parameter that showed most pronounced changes by far. It shows that monitoring of evolution of muscle fiber CSA (either fiber type specific or mean fiber CSA) can be very valuable to effectively underpin results obtained with, for example, “omics” studies. Of course, for that purpose, it is important to know which physiological meaning can be assigned to either an increase or a decrease of fiber CSA.

Essén-Gustavsson and Lindholm reported that the training-induced decrease in mean CSA in the m. gluteus medius was associated with increased citrate synthase activity (72). In a follow-up study, they reported a decrease in mean CSA in the m. vastus lateralis, to be associated with an increased oxidative and decreased glycolytic capacity (61). A smaller mean CSA in response to training is thought to be associated with a reduced glycogen storage capacity (18), and occurrence of muscle hyperplasia (muscle growth), as previously shown in Friesian horses subjected to aquatraining and dry treadmill training (73). In that study, it was shown that these morphological changes also coincide with an increased capacity to process alternative fuels such as branched-chain amino acids, aromatic amino acids, and gut microbiome-derived xenobiotics, which can more easily reach the muscular metabolic machinery, because of a smaller fiber type specific CSA. More research is needed with that respect.

The fact that no significant effect of harness training was seen on the m. pectoralis is not that surprising, since muscle kinematic studies clearly show that during trotting, the hind limb muscles show the greatest activity, creating propulsion (74). Eight weeks of dry treadmill training, another type of training, induces important changes in the m. pectoralis of Friesian horses, both from a histological and from an untargeted metabolomics point of view (22, 75–77). Dry treadmill training increases the muscle mass of the m. pectoralis, assessed by means of ultrasound, and induces a shift toward a more aerobic profile, which was further confirmed by the increase in type I and a decrease in type IIX fibers, together with a decrease in mean CSA. It seems that dry treadmill training challenges the m. pectoralis, whereas trotter harness training has a major impact on the m. vastus lateralis. This knowledge needs to be taken into account when designing muscle-specific training programs and optimizing the breed- and discipline-specific management of equine athletes. On another note, it is important to keep in mind that despite the absence of a detectable change in fiber type composition as seen in the m. pectoralis and the m. vastus lateralis in response to training in the current study, alterations at the metabolic level may have already occurred. In line with this, Hodgson et al. did not observe changes in fiber type composition of the m. gluteus medius of Thoroughbred horses after up to 12 weeks of race training, but they did find a clear upregulation of the oxidative metabolism after 6 weeks of training, as indicated by an increased CS enzyme activity (78).

In the current study, we assessed the minimal Feret's diameter that has been applied in various human and rodent muscular studies (79–86) and few equine studies (87–89). It is interesting to notice that on both occasions (fiber type specific and overall minimum Feret's diameter), different results were obtained when compared to the fiber specific and mean CSA (frame and mathematical approach). In the m. semitendinosus, a decreased CSA of the type IIAX fibers vs. a decrease of the type I minimum Feret's diameter was obtained. Furthermore, both mean frame and mean mathematical CSA showed no changes in answer to training, whereas the minimum Feret's diameter approach showed a training-induced decrease across all muscle fiber types. It would be interesting to further examine the added value of involving the minimum Feret's diameter approach in future training studies.

In the m. vastus lateralis, which showed the most overall pronounced harness training-induced adaptation in comparison to the other two muscle groups, besides a decrease in fiber type specific CSA of all muscle fiber types and a decrease in overall mean CSA for both approaches (mathematical and frame CSA), this was accompanied by a significant increase in capillary supply. No changes in mitochondrial density were detected.

In the past, it was believed that only aerobic exercise could improve capillarization in order to bring oxygen to the muscle fibers and remove waste products. It is clear that also anaerobic exercise improves capillarization through angiogenesis (90, 91). In the current study, capillary density was significantly increased in the m. vastus lateralis after 8 weeks of harness training and the number of capillaries surrounding both type I and type IIA fibers was significantly increased (Table 4). This is in accordance with a study of Chanda and coworkers who showed an increased capillary density in polo ponies subjected to 22 weeks of low-to-moderate intensity exercise and polo competitions (29). However, looking at capillary density *per se* is not enough because it does not confirm anything with respect to angiogenesis if C/F remains unchanged. Therefore, using the C/F ratio is advisable since it takes the muscle fiber size into account and thus reflects true changes in the capillary bed. In our study, C/F increased with training and, combined with a decreased mean fiber CSA and an increased capillary density, this indicates that 8 weeks of harness training induced angiogenesis. This is in agreement with a study of Serrano et al., in which Andalusian horses were subjected to 8 months of endurance training followed by 3 months of detraining. In that study, capillary density increased in the m. gluteus medius after 8 months of training and returned to baseline after 3 months of detraining (92). The same conclusions were drawn by Rivero et al., who looked into the effect of different training protocols on muscle capillarization and concluded that all training regimens (respectively 5, 15, and 25 min at speeds at blood lactate concentrations of 2.5 and 4 mmol/L for 22 days) significantly increased mean capillary number in the m. gluteus medius, whereas C/F only increased in training protocols of 15 and 25 min, respectively, but not 5 min, which means capillary changes occur only after a minimal duration and load of training (31). When comparing the increase in capillary density of type I and type IIA fibers, respectively, the increase in capillaries surrounding type IIA fibers (1.21-fold) showed a more pronounced trend when compared with type I fibers (1.17-fold) (*p* = 0.10), suggesting that capillarization targets more type IIA fibers with the applied training protocol.

In most species, baseline mitochondrial density is higher in type I fibers when compared to type IIA fibers and the subsarcolemmal region is richer in mitochondria when compared to the intermyofibrillar region (44, 93–95). This was also seen in the current study, where mitochondrial density was higher in type I fibers. Besides that, baseline mitochondrial density was significantly higher for the SS population when compared to the IMF population, in both fiber types of the m. vastus lateralis (Table 6).

In the current study, no training-induced shifts in mitochondrial density were observed in the m. vastus lateralis (Table 5). Also, other studies have reported such results, though those studies involved additional parameters to assess mitochondrial functional activity and they did, for example, find increased mitochondrial respiration despite lack of changes in histological mitochondrial density (46, 52). It is well known that early adaptations occur at the mitochondrial gene level in answer to exercise (96, 97), whereas phenotypic mitochondrial changes can occur at a later stage (36, 51). Exercise upregulates mitochondrial-related genes, such as genes involved in mitochondrial biogenesis, as has been shown in a recent study (98). In that study, it was also shown that gut microbiome composition was associated with mitochondrial gene expression in race horses, a finding that underlines the importance of the gut–muscle axis (96, 98–100). A possible explanation for the lack of change in mitochondrial density seen in the current training study could be that, in horses, the first steps of physiological training adaptation are reflected by early metabolic changes and gene expression, followed by changes in capillarization and shifts in muscle fiber CSA, prior to increases in mitochondrial density. This would be the preferential scenario for an accelerated supply of, for example, gut-derived xenobiotics to the muscular oxidative machinery.

The observed training-induced changes in the current study suggest that the muscular machinery models itself toward an optimal smaller individual muscle fiber structure to receive and process fuels that can be swiftly delivered by the circulatory system and do not require a major transformation of the mitochondrial machinery. In a previous training study, we have shown that branched-chain amino acids, aromatic amino acids, and gut microbiome-related xenobiotics function as important fuels (73). These metabolites also showed remarkable changes in other previous training studies, however, without receiving further attention (40, 101–103). Most probably, these fuels are processed in both type I and type IIA fibers. Unlike glycogen and fatty acids, these fuels are not stored within muscle fibers and need a rapid gut–muscle gateway to reach these fibers. It is more than feasible that the circulatory system may act as a conduit to provide a continuous flow of these gut microbiome-derived metabolites toward the trained muscle fibers. As a consequence, the storage of fuels is unnecessary because the resource is inexhaustible and transport is ensured, and the stress on the mitochondrial system is modest since these fuels can feed the TCA cycle at steps further downstream from acetyl-CoA. More research is needed with that respect since this knowledge has an important impact on existing paradigms in training and feeding management.

## Conclusions

This is the first training study to involve four muscle plasticity parameters in three muscles with a different function (posture vs. locomotion). Though no baseline (pre-training) fiber type specific CSA and overall mean CSA differences could be detected between all muscle groups, there was a clear decrease in fiber type specific CSA, and this was most pronounced for the m. vastus lateralis and was accompanied by a clear increase in capillary supply. The two other muscle plasticity parameters, shifts in fiber type composition and mitochondrial density, showed little to no training-induced changes. Therefore, it will be interesting to involve both muscle fiber CSA and capillary supply in follow-up harness training studies that involve “omics” analysis techniques, which are deemed to be very sensitive, but difficult to link to solid physiological deductions, without involvement of conventional techniques.

## Data Availability Statement

The original contributions presented in the study are included in the article/supplementary material, further inquiries can be directed to the corresponding author/s.

## Ethics Statement

The animal study was reviewed and approved by Animal Ethics Committee of the Ghent University (EC 2016/40).

## Author Contributions

CM, BB, LM, and CD were responsible for the conception and design of the research. CM, BB, CV, LM, MO, and CD performed the experiments. CM, BB, LM, CV, CD, and KG performed the analyses. CM, BB, LM, CV, WD, LL, and CD interpreted the results. CM, BB, CV, and CD drafted the manuscript. CM, BB, MO, LL, WD, KG, and CD edited and revised the manuscript. All authors read and approved the final version of the manuscript and accountable for all aspects of the work.

## Funding

This study was partly funded by the Fonds Wetenschappelijk Onderzoek (FWO) Flanders, Belgium (grant number 1S57617N). The experiments were performed at the Department of Translational Physiology, Infectiology and Public Health. Research Group of Comparative Physiology, Faculty of Veterinary Medicine, Ghent University, Belgium and at a training facility (EC 2016/40).

## Conflict of Interest

The authors declare that the research was conducted in the absence of any commercial or financial relationships that could be construed as a potential conflict of interest.

## Publisher's Note

All claims expressed in this article are solely those of the authors and do not necessarily represent those of their affiliated organizations, or those of the publisher, the editors and the reviewers. Any product that may be evaluated in this article, or claim that may be made by its manufacturer, is not guaranteed or endorsed by the publisher.
